# Integrated Analysis of Genes Associated With Immune Microenvironment and Distant Metastasis in Uveal Melanoma

**DOI:** 10.3389/fcell.2022.874839

**Published:** 2022-03-30

**Authors:** Wenchuan Zhou, Jing Li

**Affiliations:** Department of Ophthalmology, Xinhua Hospital Affiliated to Shanghai Jiao Tong University School of Medicine, Shanghai, China

**Keywords:** uveal melanoma, weighted gene co-expression network analysis, prognostic model, inflammatory microenvironment, metastasis

## Abstract

Inflammatory infiltration plays an essential role in the progression of tumor malignancy. The aim of this study was to identify genes associated with inflammatory microenvironment and clinical traits for survival prediction of uveal melanoma (UVM) patients. The datasets and clinical characteristics of UVM were obtained from The Cancer Genome Atlas (TCGA) and Gene Expression Omnibus (GEO) databases. We divided the UVM patients into low and high immune cell infiltration groups, identified differentially expressed genes (DEGs), constructed weighted gene co-expression network, and established prognostic prediction model and nomogram for UVM. Our analysis showed that DEGs were enriched in cytokine signaling in immune system, positive regulation of immune response and adaptive immune system. A total of fifteen candidate genes were extracted from DEGs and genes that were positively associated with tumor metastasis. Subsequently, five prognostic genes were selected to construct the final prognostic prediction model, including two up-regulated genes LHFPL3 antisense RNA 1 (LHFPL3-AS1) and LYN proto-oncogene (LYN), and three down-regulated genes SLCO4A1 antisense RNA 1 (SLCO4A1-AS1), Zinc-α2-glycoprotein 1 (AZGP1) and Deleted in Liver Cancer-1 (DLC1) in the high risk group. The model showed an Area Under Curve (AUC) value of 0.877. Our analysis highlighted the importance of immune-related genes in the progression of UVM and also provided potential targets for the immunotherapy of UVM.

## Introduction

Uveal melanoma (UVM) is the most common primary intraocular tumor in adults with an incidence of 5.1 per million in the United States (Singh et al., 2011). It is a malignant tumor that appears in the iris, ciliary body and choroid (Yang et al., 2018). Tumor metastases occur in about fifty percent of UVM patients, resulting in poor long-term survival (Kujala et al., 2003). Unfortunately, there is no effective treatment to reduce the risk of metastasis and improve overall survival (OS) of UVM patients (Damato and Coupland, 2008; Skalicky et al., 2008). Factors such as tumor basal diameter, distant metastasis, chromosome aberrations and gene expression profile, have been identified as prognostic parameters in UVM patients (Laver et al., 2010). However, the association between inflammatory phenotype and prognosis of UVM has yet been clarified. Accumulating evidence indicates that inflammatory infiltration plays an essential role in the progression of tumor malignancy. The infiltrating antigen-presenting cells (APCs) stimulate antitumor immune responses, while the infiltrating macrophages and T cells contribute to poor survival of tumor patients (de la Cruz et al., 1990; Whelchel et al., 1993; Mäkitie et al., 2001). Whether the immunological characteristics of tumor microenvironment (TME) is predictive of UVM patient survival remains unknown.

In this study, we used UVM datasets from The Cancer Genome Atlas (TCGA) and Gene Expression Omnibus (GEO) databases, and performed single-sample gene set enrichment analysis (ssGSEA) and weighted gene co-expression network analysis (WGCNA) to identify survival-related genes associated with inflammatory phenotypes and clinical traits. Subsequently, we constructed a 5-gene prognostic model based on univariate Cox regression and least absolute shrinkage and selection operator (LASSO) multivariate Cox analysis. Furthermore, we established a prognostic nomogram with four variables, namely, age, gender, metastasis and prognostic gene risk score for UVM, which could be applicable for predicting the long-term survival of UVM patients.

## Materials and Methods

### Data Sources

Two UVM cohorts were used in this study: one from GEO (GSE22138) and the other from TCGA database (TCGA_UVM). GSE22138 included 63 UVM samples obtained by enucleation in untreated patients. This dataset consists of molecular profiles derived from gene expression microarrays, identifying genes associated with metastasis in UVM. The platform used to obtain these data was GPL570 (Affymetrix Human Genome U133 Plus 2.0 Array). TCGA_UVM included 80 UVM tissue samples derived from adult patients with clinical traits. This dataset consists of divergent genomic aberrations, transcriptional features and clinical outcomes.

### Cluster of UVM Patients Based on ssGSEA Score

We obtained 29 immune-associated gene sets that represented diverse immune cell types, functions and pathways in accordance with previous literature (He et al., 2018). The ssGSEA score was applied to quantify the enrichment levels of these gene sets in each sample of the GSE22138 and TCGA_UVM cohorts using R package “GSVA” (Hänzelmann et al., 2013). According to hierarchical clustering algorithm and the results of ssGSEA, the UVM patients were assigned into two clusters: high and low immune cell infiltration. In addition, the ESTIMATE Score, Immune Score, Stromal Score and the ratio of Tumor Purity were calculated using the R package “ESTIMATE.” This package was designed to evaluate the infiltration landscape of immune and stromal cells within the TME based on gene expression profile (Yoshihara et al., 2013).

### Identification of Differentially Expressed Genes Between Immune Subtypes and Analysis of Gene Functional Enrichment

The DEGs (|log_2_Fold Change| > 1 and adjusted *p*-value <0.05) were identified between high and low immune cell infiltration clusters of both UVM cohorts as described above using R package “limma.” The 224 overlapped DEGs within these two datasets were extracted by the Venn diagram. Metascape (http://metascape.org) was used for functional enrichment analyses of the overlapped DEGs, including Gene Ontology (GO) and Kyoto Encyclopedia of Genes and Genomes (KEGG) enrichment analyses. We collected and grouped the terms with an enrichment factor >1.5, minimum count of 3, a *p* value < 0.01 into clusters.

### Construction of the Gene Co-Expression Network and Module-Trait Relationship

The 5,000 most variably expressed genes based on median absolute deviation (MAD) from GSE22138 cohort were selected for downstream co-expression analysis using R package “WGCNA” (Langfelder and Horvath, 2008; Scardoni et al., 2009). WGCNA was performed as previously described (Zhou et al., 2021b). In this study, the soft threshold was set to 6. A total of 15 intersecting genes between overlapped DEGs above and module blue were extracted by the Venn diagram.

### Construction of the Prognostic Model and Nomogram

The univariate Cox regression analysis was performed to evaluate the prognostic value of selected genes using R package “survival”. Genes with *p*< 0.05 were considered as candidates to fit LASSO multivariate Cox analysis for UVM patients. As a result, a prognostic model based on five genes was established. According to the median risk score, we divided UVM cohort into low- and high-risk subgroups. Prognostic model and nomogram were established essentially as described in detail previously (Zhou et al., 2021a).

## Results

### Identification and Evaluation of Two Immune Subtypes in UVM

**FIGURE 1:**
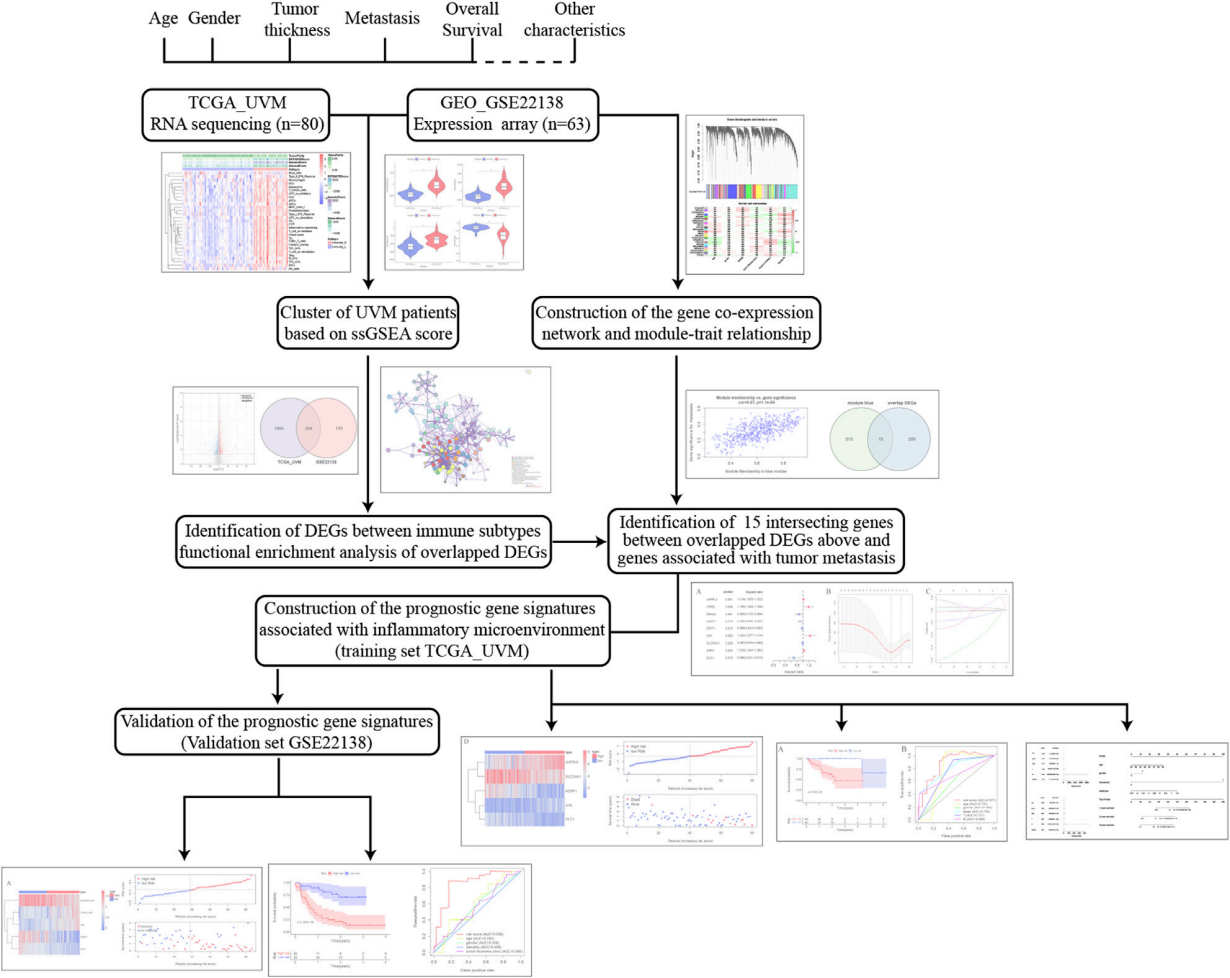
The workflow of the analysis in this study.

**FIGURE 2:**
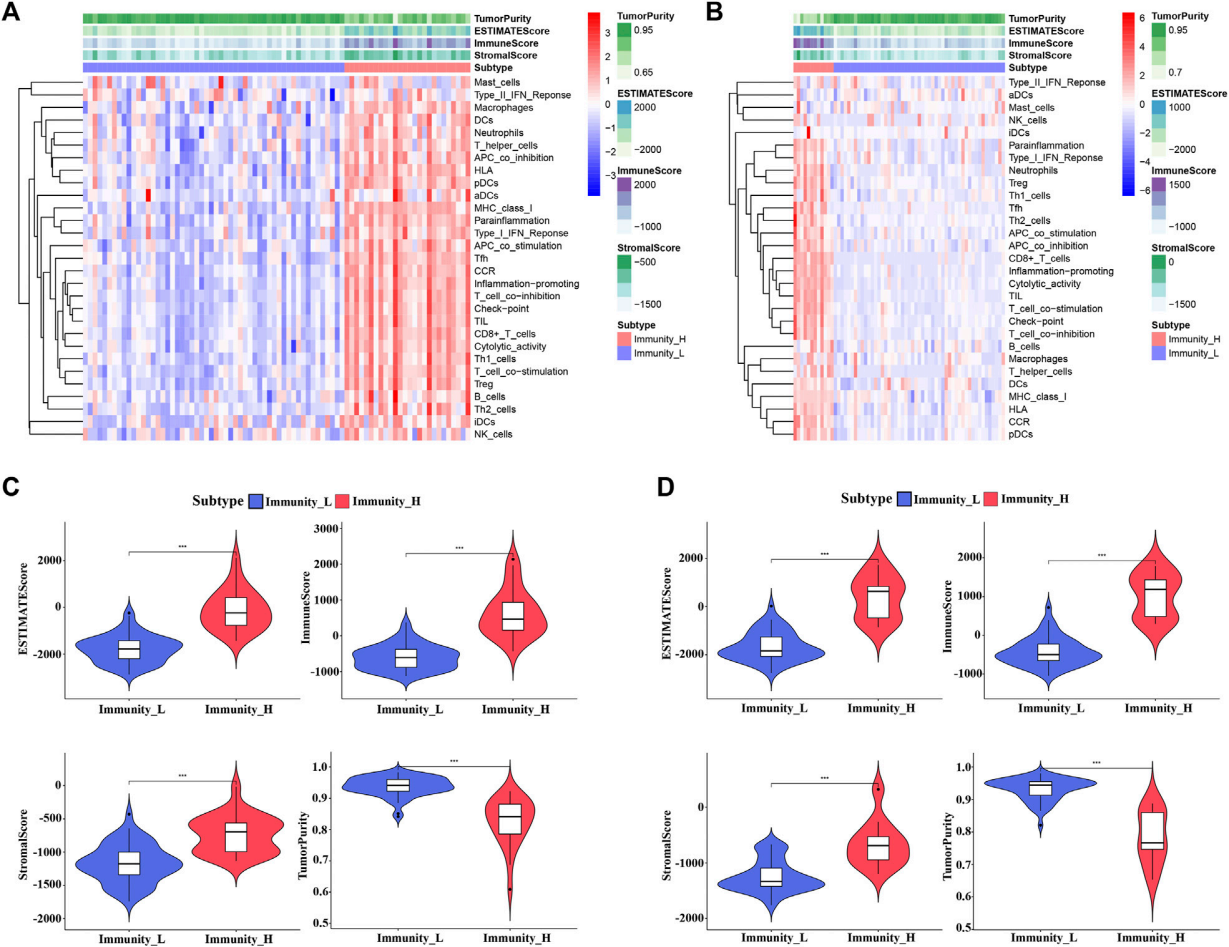
Construction and evaluation of UVM clusters. **(A,B)** The enrichment levels of 29 immune-associated gene sets in the low (Immunity_L) and high (Immunity_H) immune cell infiltration groups in TCGA_UVM cohort **(A)** and GSE22138 cohort **(B)**. **(C,D)** The violin plot showed the difference in ESTIMATE Score, Immune Score, Stromal Score, and Tumor Purity between two clusters in TCGA_UVM cohort **(C)** and GSE22138 cohort **(D)**.

The workflow of the analysis was shown in Figure 1. The enrichment levels of 29 immune signatures were quantified using the ssGSEA score in TCGA_UVM and GSE22138 cohorts (Supplementary Table S1). According to the levels of immune infiltration, we assigned the 80 UVM samples from TCGA into two clusters: the low immune cell infiltration cluster (*n* = 54) and high immune cell infiltration cluster (*n* = 26) (Figure 2A). Similarly, sixty-three UVM samples of GSE22138 were assigned into the low immune cell infiltration cluster (*n* = 51) and high immune cell infiltration cluster (*n* = 12) (Figure 2B). Subsequently, the ESTIMATE algorithm was applied to calculate the ESTIMATE Score, Immune Score, Stromal Score and the ratio of Tumor Purity (Figures 2C,D). The results showed that the ESTIMATE Score, Immune Score and Stromal Score were higher in the high immune cell infiltration cluster, while the ratio of Tumor Purity was lower in the high immune cell infiltration cluster for both TCGA_UVM and GSE22138 cohorts.

### Identification of DEGs and Analysis of Gene Functional Enrichment

**FIGURE 3:**
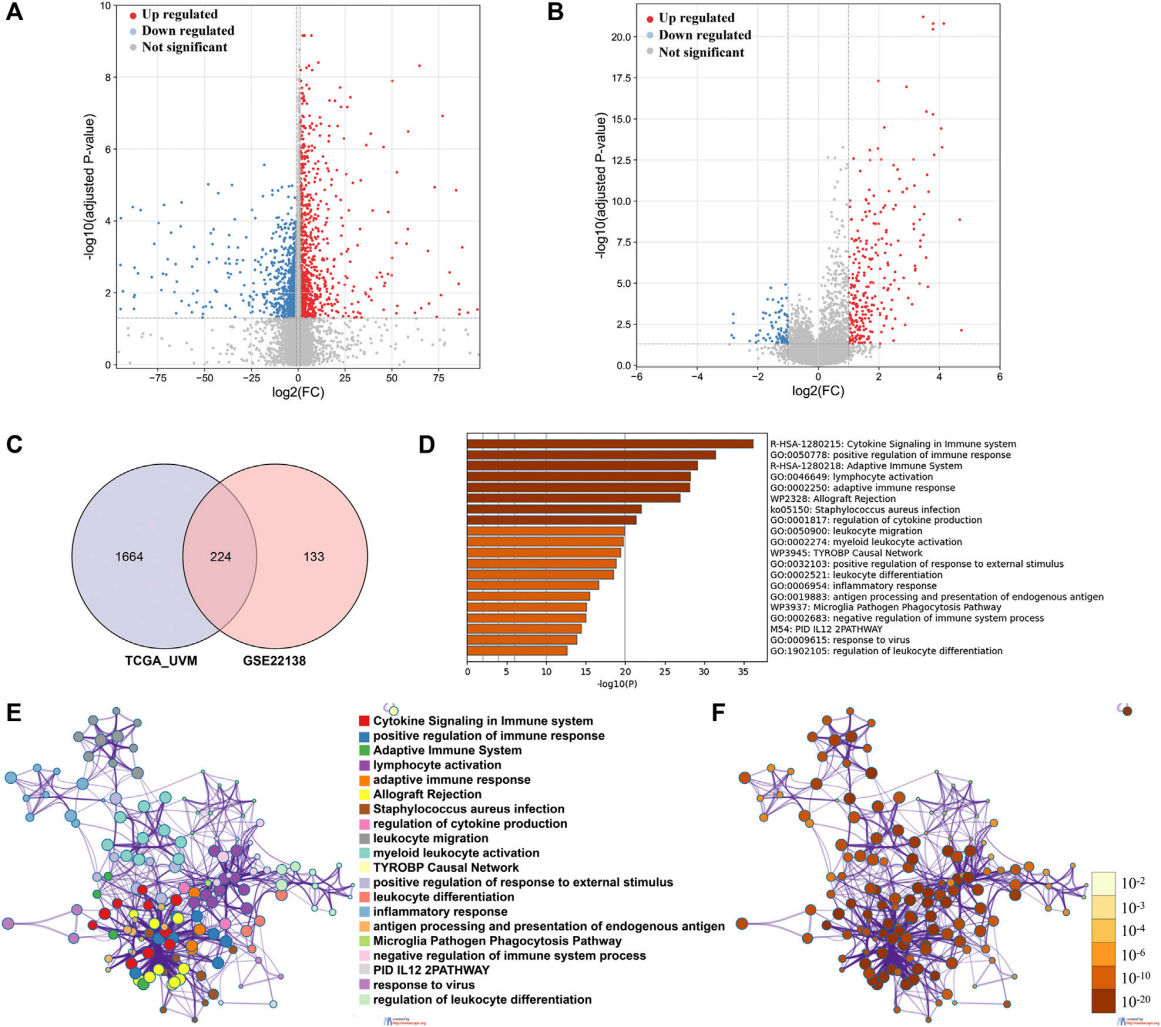
Identification of DEGs and analysis of gene functional enrichment. **(A,B)** The volcano plot showed the DEGs between low and high immune cell infiltration groups in TCGA_UVM cohort **(A)** and in GSE22138 cohort **(B)**. **(C)** Venn Diagram showing 224 overlapped DEGs between TCGA_UVM and GSE22138 cohorts. **(D)** GO terms and pathways enriched in the 224 overlapped DEGs. **(E)** Network of enriched terms colored by cluster ID, where nodes that share the same cluster ID are typically close to each other. **(F)** Network of enriched terms colored by p-value, where terms containing more genes tend to have a more significant p-value.

We identified 1888 DEGs between low and high immune cell infiltration clusters in TCGA_UVM cohort, including 960 down-regulated and 928 up-regulated genes (Figure 3A); and 357 DEGs in the GSE22138 cohort, including 83 down-regulated and 274 up-regulated genes (Figure 3B). There were 224 common genes between the sets of DEGs, including 48 down-regulated and 176 up-regulated genes (Figure 3C and Supplementary Table S2). We then performed functional enrichment analysis using Metascape on the common DEGs and found that they were enriched mainly in GO: 0050778 (positive regulation of immune response), GO: 0046649 (lymphocyte activation), GO: 0002250 (adaptive immune response), GO: 0001817 (regulation of cytokine product) and GO: 0050900 (leukocyte migration) (Figures 3D–F).

### Construction of the Co-Expression Network and Module-Trait Relationship

**FIGURE 4:**
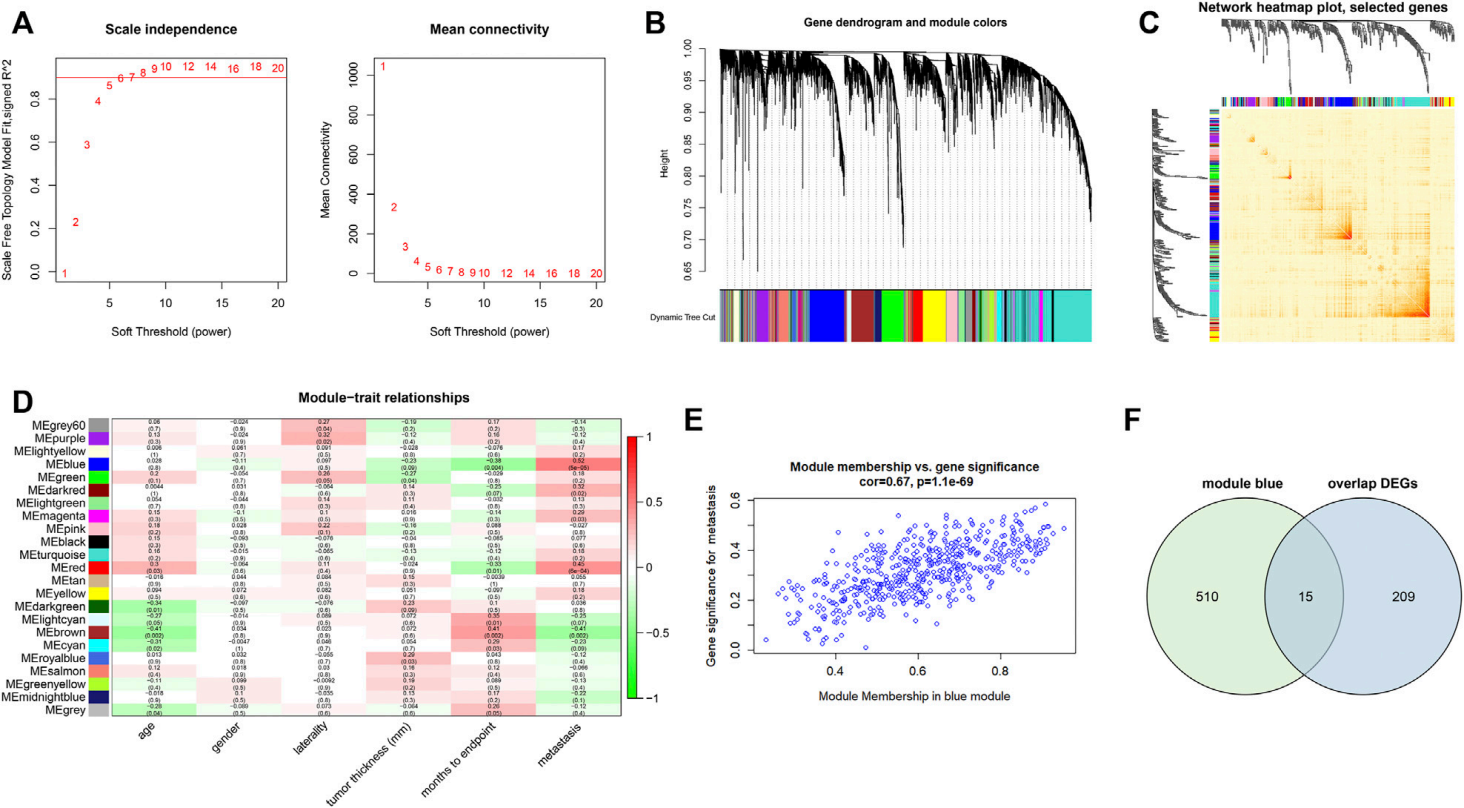
Construction of co-expression modules and module-trait relationship. **(A)** Analysis of network topology for soft threshold power values. **(B)** Hierarchical clustering dendrogram of probe sets with dissimilarity in accordance with topological overlap and assigned module colors. **(C)** Network heatmap plot with topological overlap matrix among 400 randomly selected genes. **(D)** Module-trait relationships. A total of 22 modules were identified in this study. The clinical traits included age, gender, laterality, tumor thickness, months to the endpoint and metastasis. **(E)** The scatterplots of blue module membership vs. gene significance for metastasis. **(F)** Venn diagram showing 15 intersecting genes between overlapped DEGs and blue module.

We identified 5,000 most variably expressed genes from the gene expression profiles of GSE22138 to build the co-expression network. When the power was set to 6, a scale-free network distribution was constructed between genes (Figure 4A). In this study, twenty-two co-expression modules were generated with different colors. The clinical traits included age, gender, laterality, tumor thickness, months to endpoint and metastasis (Figures 4B–D). Among these modules, the blue module was significantly associated with tumor metastasis (r = 0.67, *p* = 1.1e-69) (Figure 4E). Therefore, blue module was chosen for further analysis as module of interest. Using Venn diagram, we identified 15 common genes between DEGs and genes associated with metastasis (blue module) (Figure 4F).

### Identification of the Prognostic Gene Signature Associated With Inflammatory Microenvironment

**FIGURE 5:**
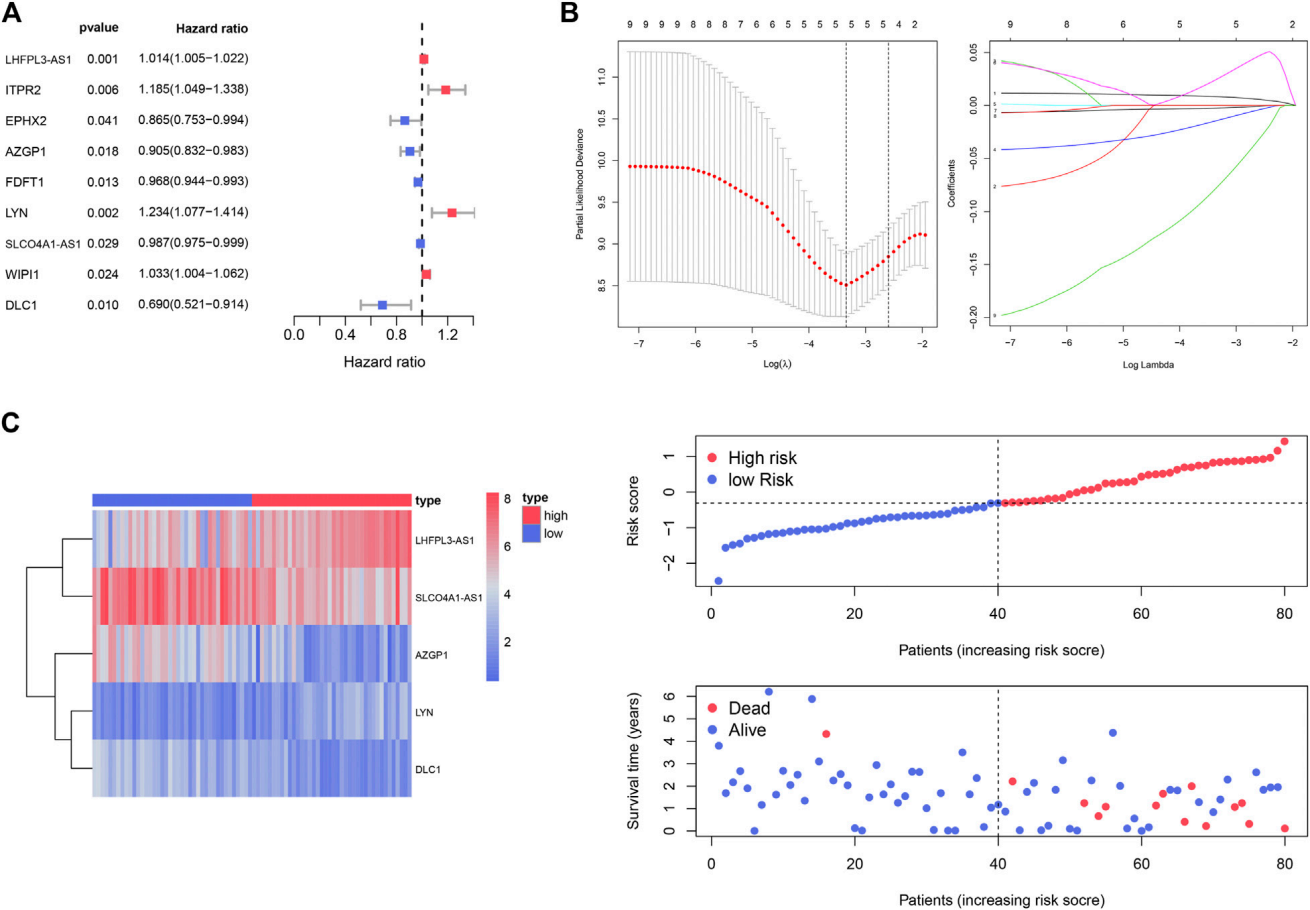
Identification of the prognostic gene signature for UVM patients in the TCGA_UVM set. **(A)** A total of nine genes were identified as survival-related genes (*p* < 0.05) using univariate Cox regression. **(B)** A prognostic model was established with five genes to predict the OS of UVM patients using LASSO multivariate Cox analysis. **(C)** UVM patients were divided into low- and high-risk subgroups based on the median cutoff risk score: the heatmap of gene expression, distribution of risk score and survival status between the two groups.

The 15 genes identified above were further evaluated using univariate Cox regression analysis, and nine genes were identified as survival-related genes (*p* < 0.05) in the TCGA_UVM cohort (Figure 5A). Subsequently, we established a prognostic model with five genes to predict the OS of UVM patients using LASSO multivariate Cox analysis (Figure 5B and Supplementary Table S3). These were LHFPL3 antisense RNA 1 (LHFPL3-AS1), SLCO4A1 antisense RNA 1 (SLCO4A1-AS1), Zinc-α2-glycoprotein 1 (AZGP1), LYN proto-oncogene (LYN) and Deleted in Liver Cancer-1 (DLC1). According to the median value of risk scores, the UVM patients of the TCGA_UVM cohort were divided into low- and high-risk groups. The differences in gene expression, distribution of risk score and survival status between the two groups are shown in Figure 5C.

**FIGURE 6:**
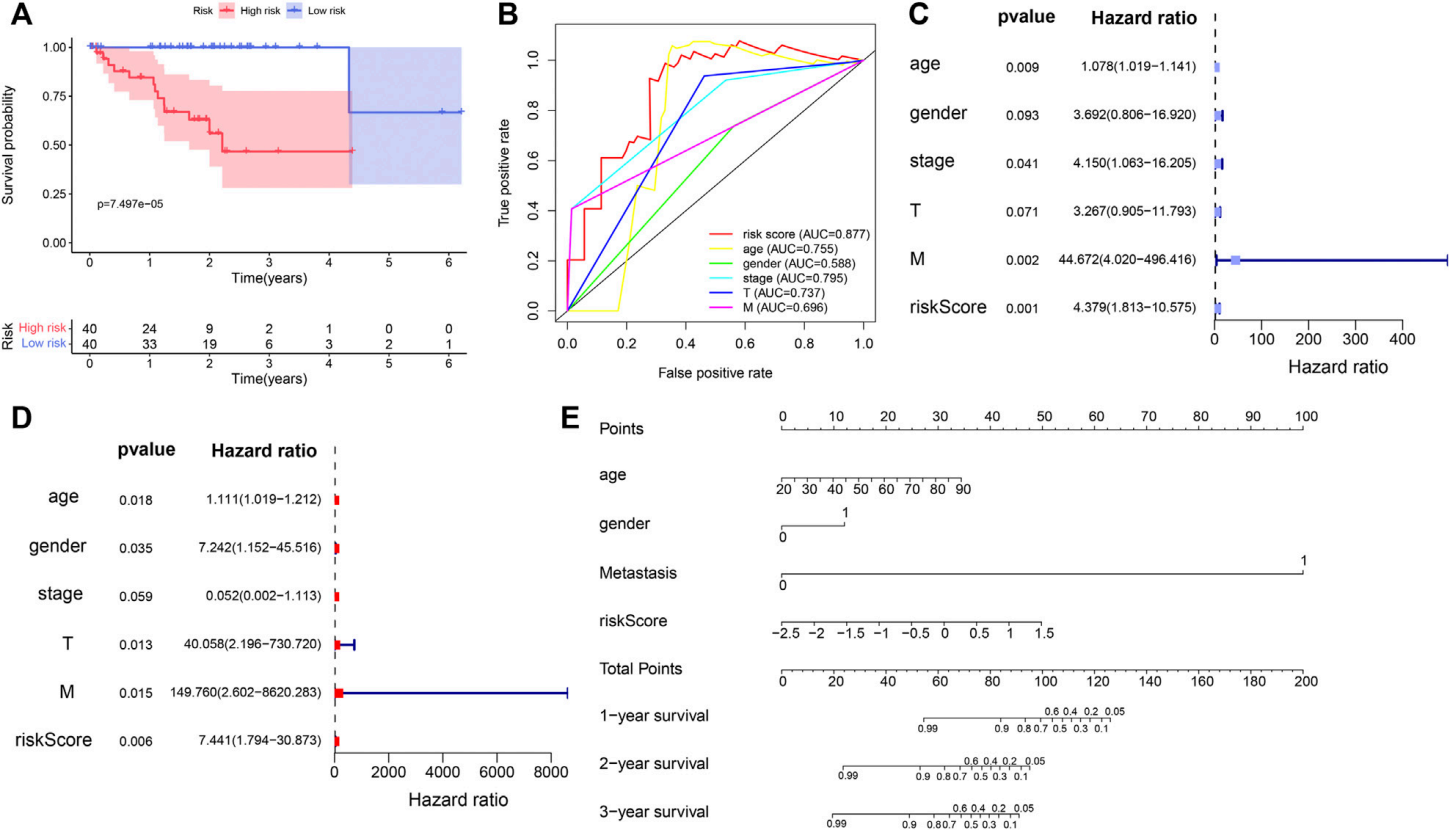
Evaluation of prognostic value of the five immune-related gene signature in the TCGA_UVM set. **(A)** Kaplan–Meier curve of UVM patients in the low- and high-risk groups of TCGA_UVM cohort. **(B)** ROC curves with calculated AUCs of the five immune-related gene signature. **(C)** Univariate cox analysis indicated that age (*p* = 0.009), stage (*p* = 0.041), metastasis (*p* = 0.002) and risk score (<0.001) were associated with the UVM prognosis. **(D)** Multivariate Cox analysis showed that risk score was confirmed as independent OS-related factor (*p* = 0.006). **(E)** The nomogram for the prediction of survival probability of UVM patients.

Our results showed that the survival time was significantly different between the two subgroups. The OS of high-risk UVM patients was significantly poorer than that of the low-risk group (*p* = 7.497e-05) (Figure 6A). In addition, the prognostic gene signature exhibited the most robust predictive efficiency, with an AUC of 0.877 (Figure 6B). The univariate Cox analysis showed that age (*p* = 0.009), stage (*p* = 0.041), metastasis (*p* = 0.002) and immune-related gene risk score (*p* = 0.001) were also associated with the UVM prognosis (Figure 6C). Multivariate Cox analysis showed that the five immune-related gene signature was independent OS-related factor (*p* = 0.006) (Figure 6D). A total of four variables, namely, age, gender, metastasis, and risk score, were incorporated into the nomogram with immune-related genes (Figure 6E).

### Validation of the Prognostic Gene Signature

**FIGURE 7:**
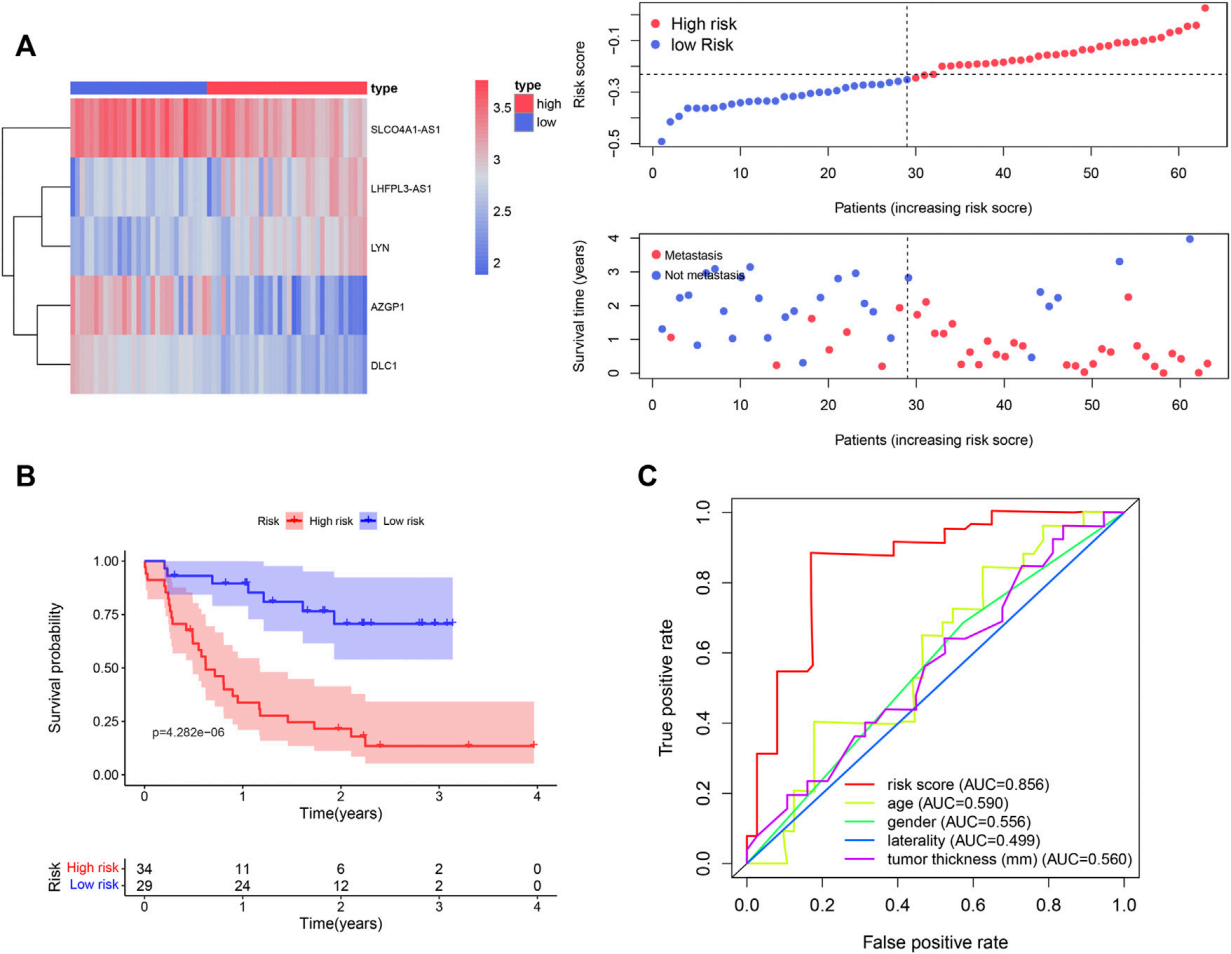
Validation of the five immune-related gene signature in the GSE22138 cohort. **(A)** A total of 63 UVM patients were divided into low- and high-risk subgroups based on the median cutoff risk score. The heatmap of gene expression, distribution of risk score and survival status between the two groups were shown. **(B)** Kaplan–Meier curve of UVM patients in the low- and high-risk groups. **(C)** ROC curves with calculated AUCs of the five immune-related gene signature.

We used the UVM patients in the GSE22138 cohort to validate the prognostic gene signature obtained above. Similar to the analysis performed on the UVM patients in TCGA_UVM, we separated the patients in GSE22138 into low- and high-risk groups according to the median risk scores of all patients in the cohort. The heatmap of gene expression, distribution of risk score and survival status between the two groups are visualized in Figure 7A. The results of Kaplan–Meier curves showed that OS of high-risk UVM patients was significantly poorer than low-risk group (*p* = 4.282e-06) (Figure 7B). Furthermore, the prognostic immune gene signatures exhibited a robust predictive efficiency, with an AUC of 0.856 (Figure 7C).

## Discussion

Immune microenvironment plays an essential role in the tumor progression. Immunological heterogeneity of the TME is associated with the prognosis of patients with various cancers (Nienhuis et al., 2015; Tao et al., 2021). Prognostic risk models consisting of TME-related features were identified in patients with glioma and lung adenocarcinoma (LUAD) (Wang et al., 2021; Zhong et al., 2021). In this study, we focused on the roles of local inflammatory microenvironment in the prognosis of UVM patients. Integrated analysis of genes associated with immune microenvironment and distant metastasis was performed using the ssGSEA score and WGCNA in the UVM samples. The survival-related genes were identified and used to construct a prognostic model that exhibited a robust predictive efficiency both in the TCGA and GEO cohorts. These genes provided potential targets for immunotherapy of UVM.

The five genes in the prognostic model included two (*LHFPL3-AS1* and *LYN*) which were up-regulated in the high risk group and three (*SLCO4A1-AS1*, *AZGP1*, and *DLC1*) which were down-regulated in the high risk group. There were studies which suggested the involvement of each of these genes in tumorigenesis. For example, *LHFPL3-AS1*, which encodes a long non-coding RNA (lncRNA), was found to promote tumorigenesis of melanoma stem cells (Zhang et al., 2020). The up-regulation of *LHFPL3-AS1* expression contributed to poor prognosis of melanoma patients (Peng et al., 2020). *LYN* encodes a tyrosine protein kinase of the Src family which is abundantly expressed in immune cells and involved in the occurrence and progression of tumors, including breast cancer, lung cancer, colorectal cancer and chronic leukemia (Wu et al., 2008; Elsberger et al., 2010; Su et al., 2012; Ahluwalia et al., 2021). The expression of Lyn kinase was also increased in melanoma tissues and cells. Results of an *in vitro* study showed that *LYN* knockdown inhibited the proliferation, migration and invasiveness of melanoma cells (Zhang et al., 2019). *AZGP1* encodes a secreted protein (zinc-binding glycoprotein) which is involved in lipid metabolism and cell cycle. Studies showed that *AZGP1* participated in the regulation of tumorigenesis, such as breast cancer, prostate cancer, liver cancer, gastric cancer, colon cancer and LUAD (Albertus et al., 2008; Dubois et al., 2010; Huang et al., 2012; Huang et al., 2013; Jung et al., 2014; Xue et al., 2014). A multicenter study focusing on the association between the expression level of *AZGP1* and the prognosis of patients with prostate cancer showed that decreased expression of *AZGP1* was related to poor prognosis and recurrence of prostate cancer (Brooks et al., 2016). Additionally, down-regulation of AZGP1 was also associated with poor prognosis in liver and gastric cancers (Huang et al., 2012; Huang et al., 2013). *DLC1* encodes a GTPase-activating protein. It functions as a tumor suppressor in various cancers, including breast, prostate, gastric and lung cancers (Kim et al., 2003; Plaumann et al., 2003; Yuan et al., 2004; Guan et al., 2006). Previous study reported the association between decreased expression of cytoplasmic DLC1 and worse OS in metastatic melanoma (Sjoestroem et al., 2014). *SLCO4A1-AS1* was found upregulated in various cancers and associated with tumor metastasis and worse OS of patients (Yu et al., 2018; Tang et al., 2019; Wu et al., 2021). However, our pooled results showed that decreased expression of this lncRNA indicated a poor prognosis of UVM patients. Further investigations on the expression of these genes in UVM patients are required to evaluate the potential therapeutic values in UVM.

Some limitations of this study should be considered. First, our study was limited by the number of tumor samples. The performance of five-gene prognostic model should be validated in a large UVM patient cohort. Although the model we proposed in this study displayed stable performance, more databases are needed to verify the accuracy. Second, the prediction model constructed in this study was based on public datasets and bioinformatic analyses. The biological functions of the immune-related genes in this model need further wet-experimental research. In addition, we highlighted the biological role of individual gene inside the model, which is consistent with its prediction role. However, molecular mechanisms of interaction between these genes should be considered as well, which need further exploration.

## Conclusion

In this study, we proposed a prognostic model consisting of five genes associated with inflammatory microenvironment and tumor metastasis to predict OS for UVM patients. Our findings highlighted the importance of immune-related gene signatures in the malignant development of UVM and provided potential targets for immunotherapy of the disease.

## Data Availability

The datasets presented in this study can be found in online repositories. The names of the repository/repositories and accession number(s) can be found in the article/Supplementary Material.
